# Supplementary figures and images for: Population genomic evidence that human and animal infections in Africa come from the same populations of Dracunculus medinensis
Source: PLoS Negl Trop Dis. 2020 Nov 30;14(11):e0008623. doi: 10.1371/journal.pntd.0008623 (PMC7728184; doi:10.1371/journal.pntd.0008623)

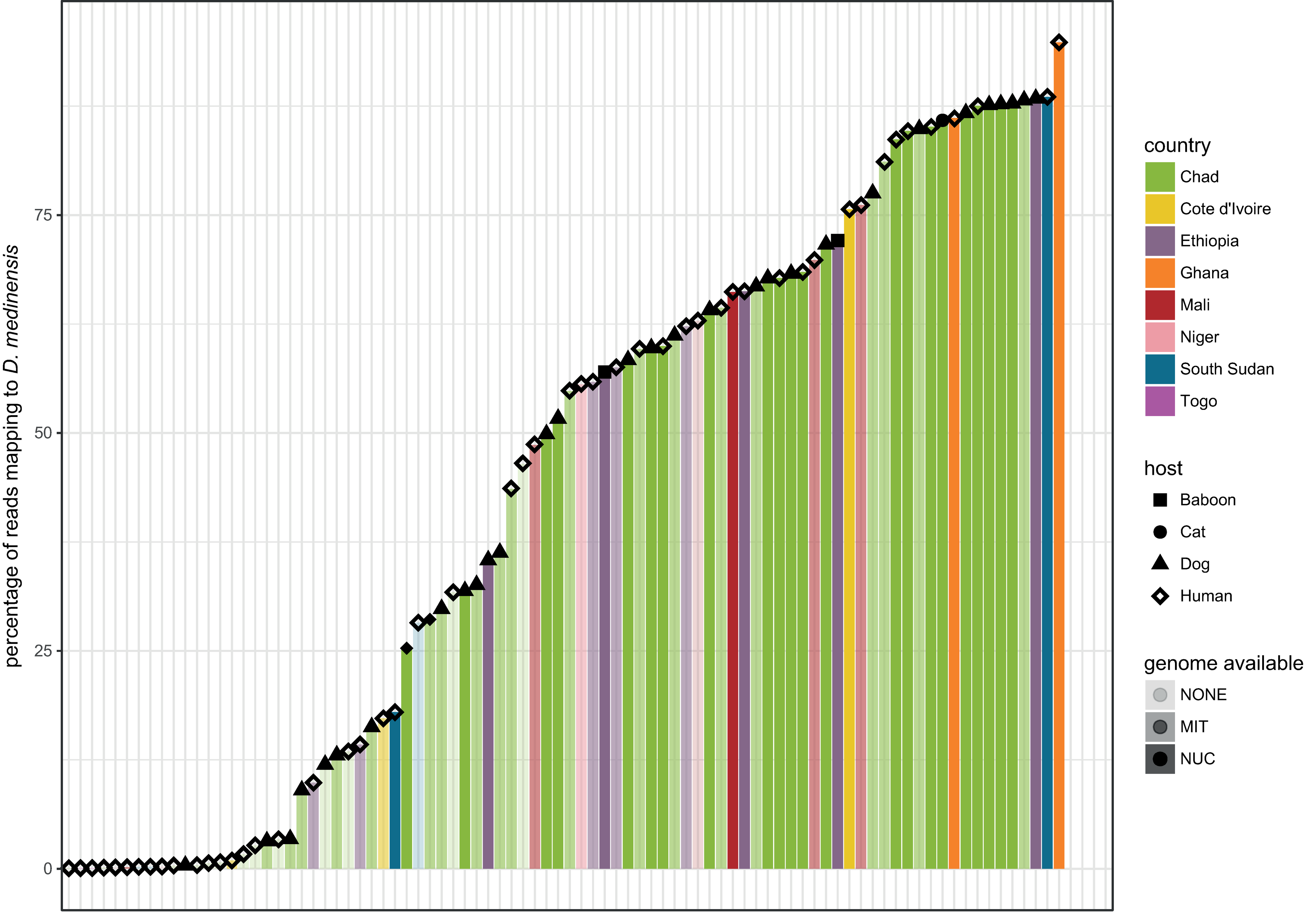

Supplement: S1 Fig — Each bar indicates the proportion of sequencing reads from each sample that mapped against the reference genome assembly. The density of each bar indicates whether whole-genome data is included in our analysis, only mitochondrial genome data or whether insufficient data was available for that sample. (TIF) [file pntd.0008623.s001.tif]

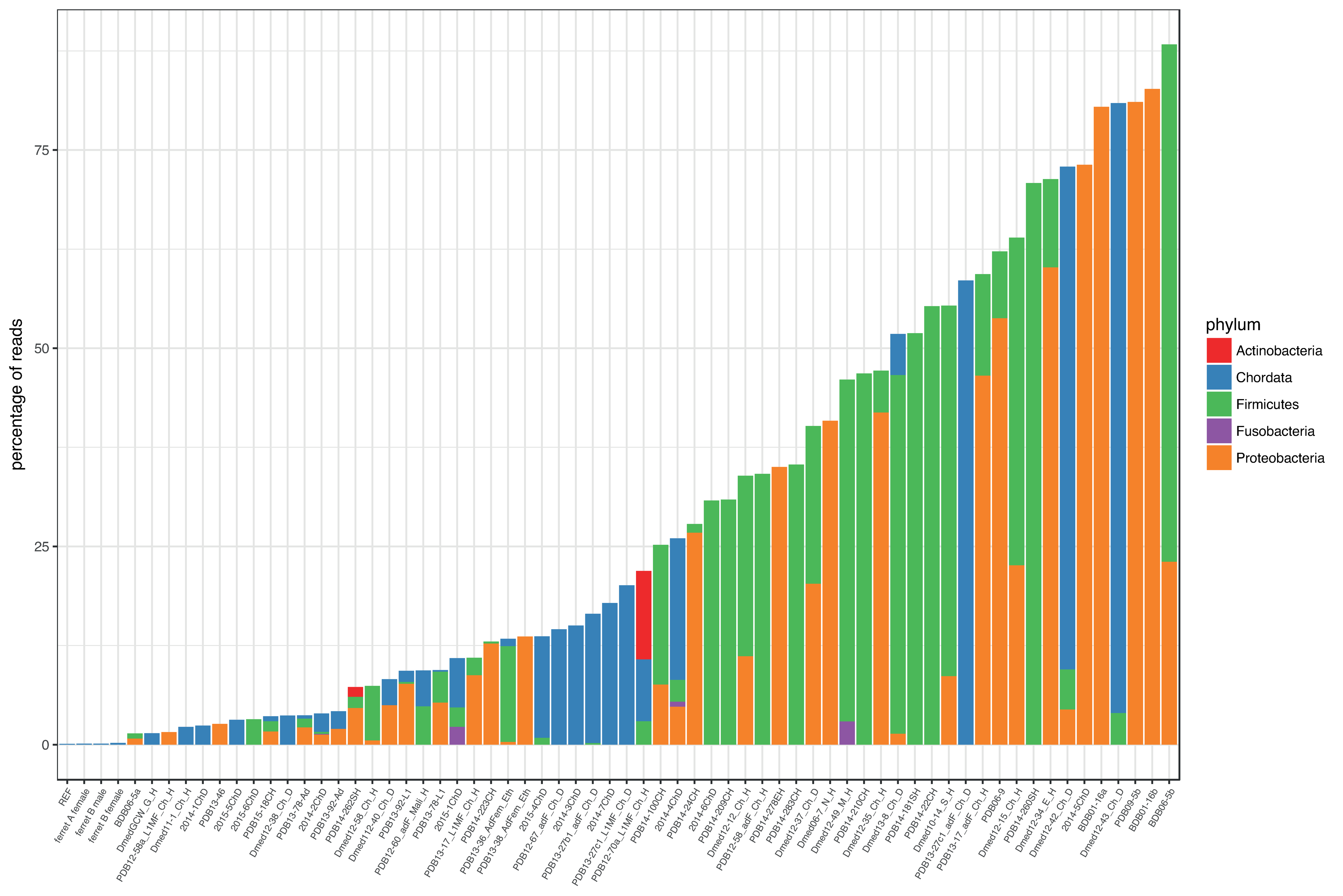

Supplement: S2 Fig — Number of reads inferred by k-mer analysis to originate from different phyla. Data are shown for all phyla to which at least 50,000 reads were assigned. 68 samples are shown: those not shown here did not match any phyla with his cut-off. Note that no nematode sequences are in the database used for this search (see Methods). (TIF) [file pntd.0008623.s002.tif]

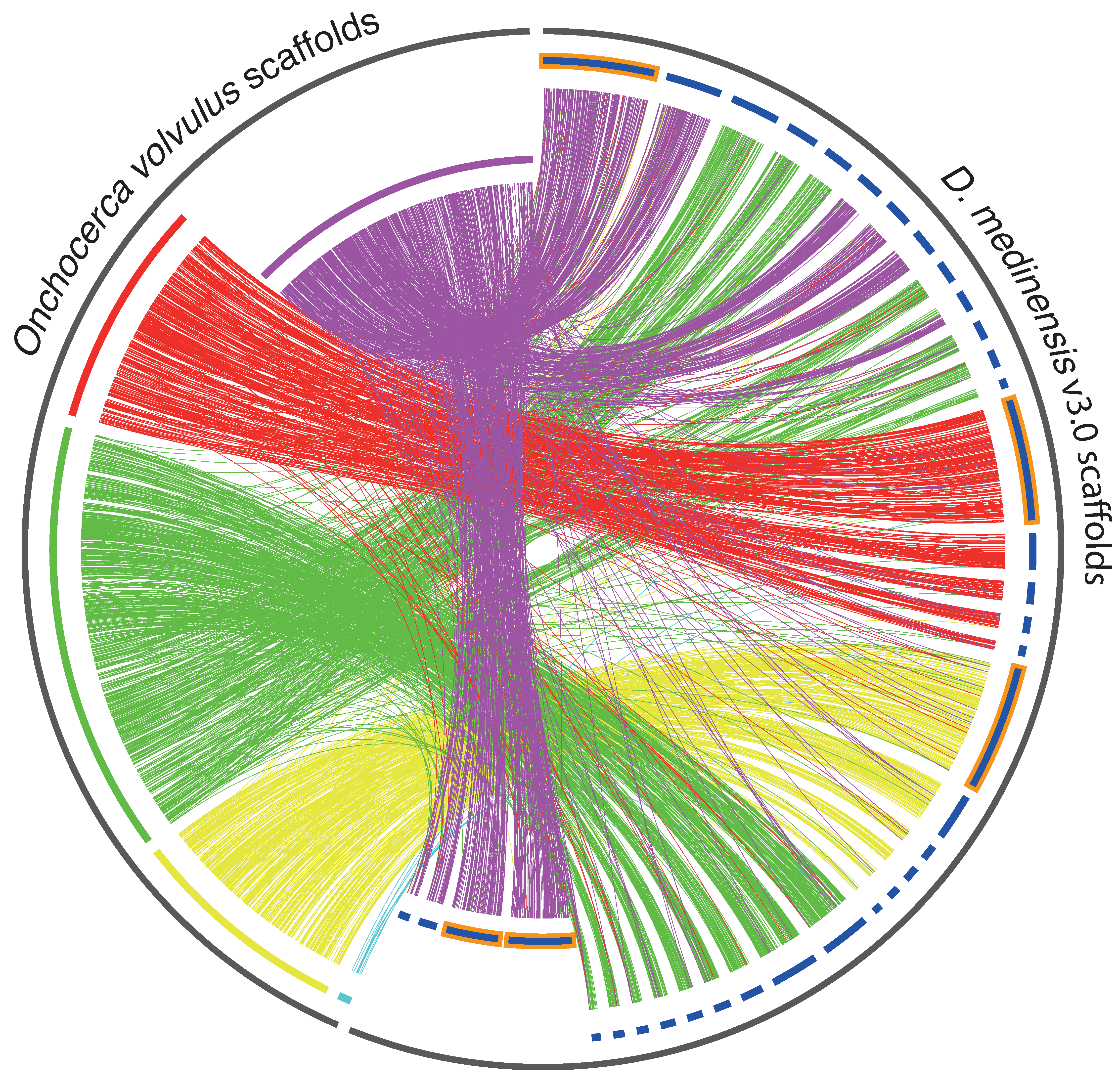

Supplement: S3 Fig — Lines connect sequences for which the conceptual amino acid translations are at least 50% identical over 250 amino acids. D. medinensis scaffolds highlighted in orange are those shown in Fig 2A. Note that one of the longest scaffolds matches to the opposite end of the X-chromosome scaffold in O. volvulus to the scaffolds with reduced coverage in male worms. This region of O. volvulus X was not part of the ancestral filarial X chromosome (Cotton et al., 2016), and so is not expected to be part of D. medinensis X and is thus labelled as autosomal in Fig 2A, and considered as autosomal in our analyses here. D. medinensis scaffolds with reduced male coverage are shown inset. (TIF) [file pntd.0008623.s003.tif]

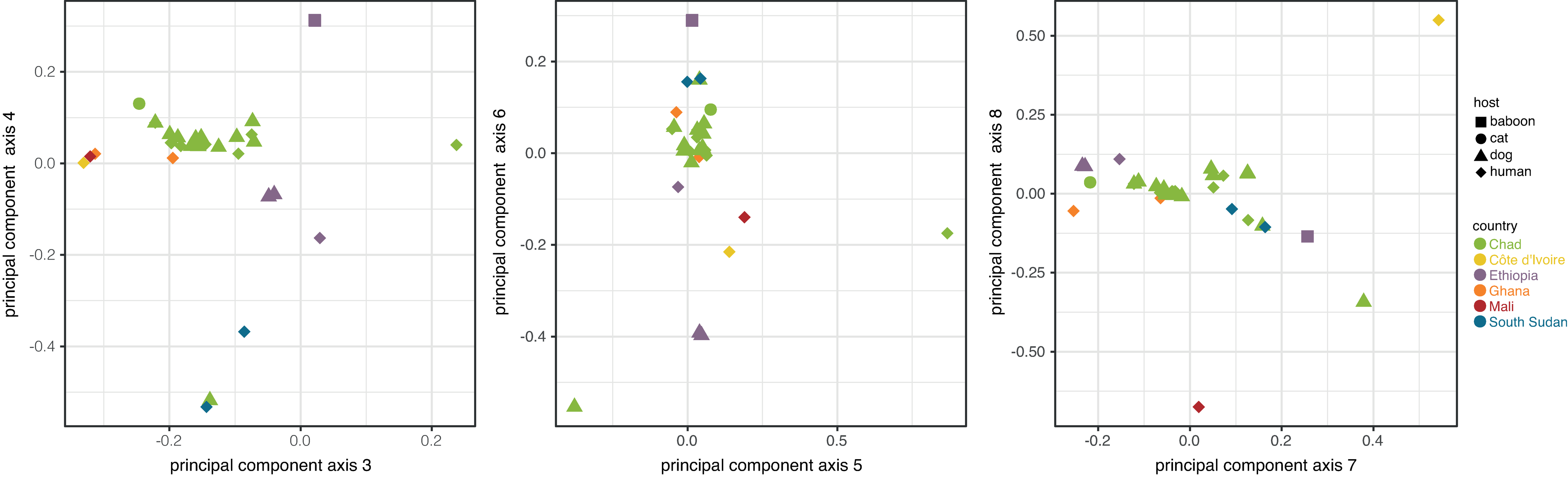

Supplement: S4 Fig — (TIF) [file pntd.0008623.s004.tif]

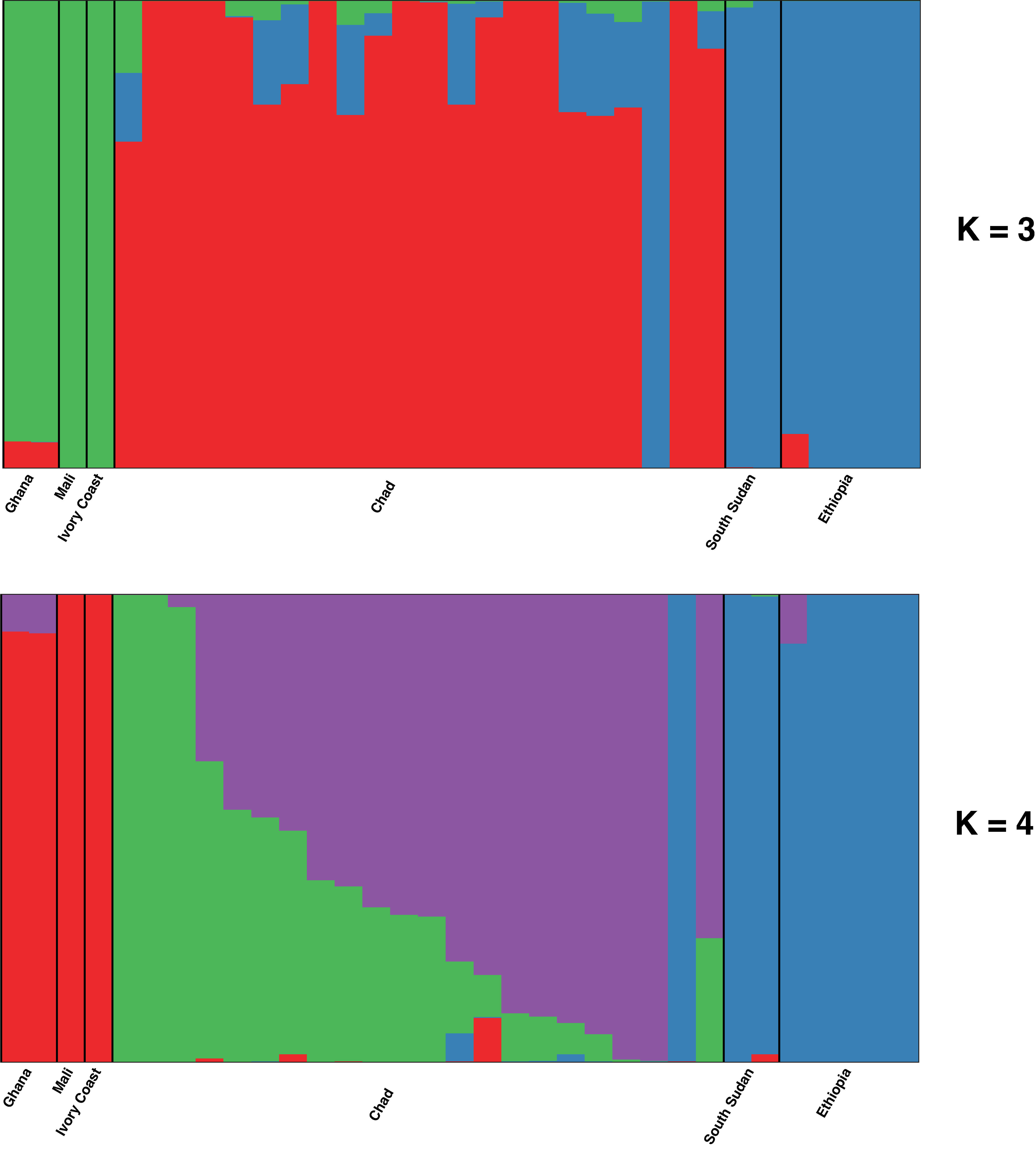

Supplement: S5 Fig — Vertical bars represent individuals, with the proportion of each color in each bar representing the proportion of inferred ancestry of that individual from the population. Shapes and colours of symbols along the x-axis indicate host and country worms were collected from. (TIF) [file pntd.0008623.s005.tif]

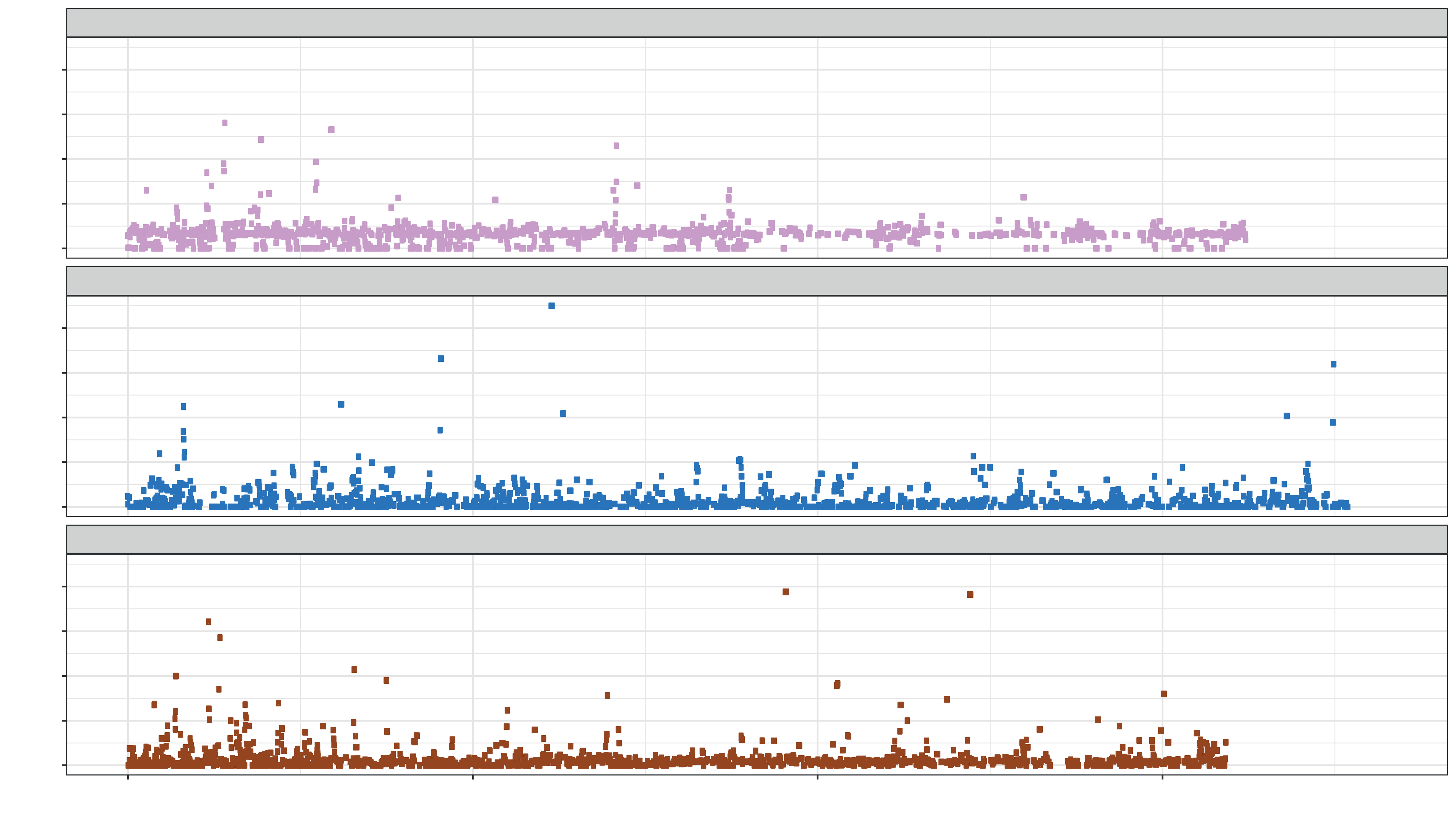

Supplement: S6 Fig — Values shown are mean Fst for non-overlapping 1kb windows centered at the position shown on the x-axis. (TIF) [file pntd.0008623.s006.tif]

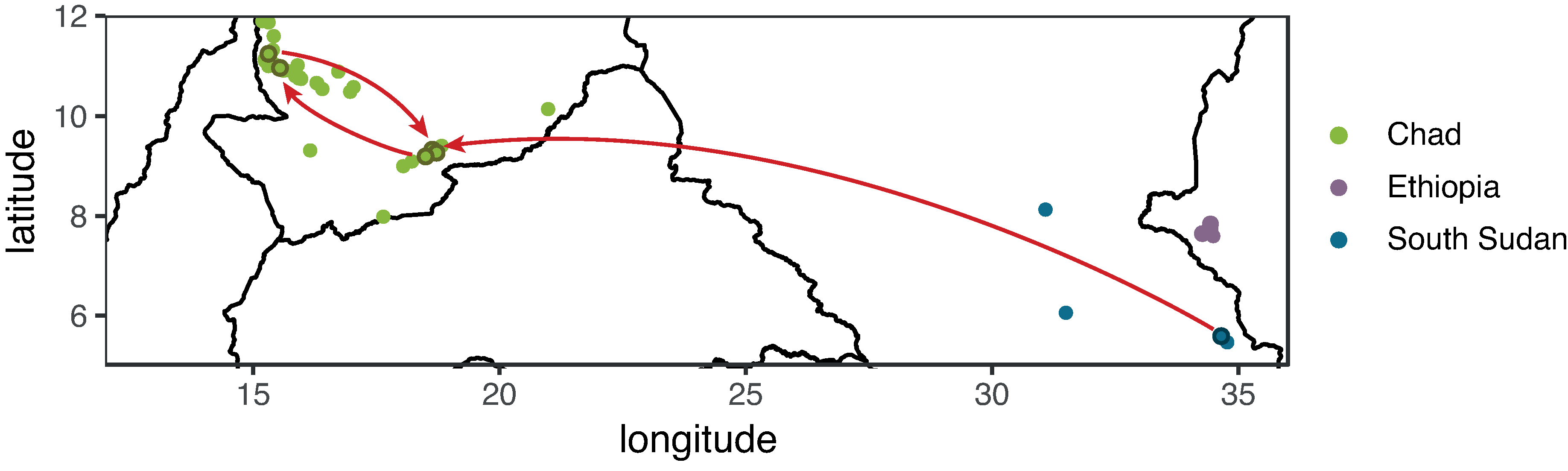

Supplement: S7 Fig — Sample locations are indicated by dots, colour-coded by country of isolation. Red arrows indicate inferred parent-offspring relationships between samples; samples involved in these links are highlighted by dark rings around the point at which the infection was detected. The locations of detection may not represent the locations at which infections were acquired, or the location of residence of the hosts. (TIF) [file pntd.0008623.s007.tif]
