# Supplementary material for: Population genomic evidence that human and animal infections in Africa come from the same populations of Dracunculus medinensis
Source: PLoS Negl Trop Dis. 2020 Nov 30;14(11):e0008623. doi: 10.1371/journal.pntd.0008623 (PMC7728184; doi:10.1371/journal.pntd.0008623)
Supplement: S1 Table — Note that mean and median coverage are defined over the whole nuclear genome assembly for both MIT and NUC samples. Reads and mapping statistics are for the sum across all sequenced libraries and lanes. ENA = European Nucleotide Archive. (DOCX) [file pntd.0008623.s008.docx]

| sample name | country | host | variant data | total reads | reads mapping | percent mapping | mean coverage | median coverage | number of lanes | ENA accession numbers |
| --- | --- | --- | --- | --- | --- | --- | --- | --- | --- | --- |
| PDB14-138CH | Chad | Human | no library made |  |  |  |  |  |  |  |
| PDB14-23CH | Chad | Human | no library made |  |  |  |  |  |  |  |
| PDB14-25CH | Chad | Human | no library made |  |  |  |  |  |  |  |
| PDB14-287MH | Mali | Human | no library made |  |  |  |  |  |  |  |
| BDB06-5b | Cote d'Ivoire | Human | MIT | 2,229,520 | 21,157 | 0.95 | 0.03 | 0 | 1 | ERR460382 |
| PDB06-9 | Cote d'Ivoire | Human | MIT | 2,115,778 | 365,211 | 17.26 | 0.53 | 0 | 1 | ERR460383 |
| PDB14-181SH | South Sudan | Human | NONE | 228,914 | 64,654 | 28.24 | 0.09 | 0 | 1 | ERR1081345 |
| PDB14-260SH | South Sudan | Human | NONE | 1,095,228 | 2,610 | 0.24 | 0.00 | 0 | 2 | ERR1081343 |
| PDB14-262SH | South Sudan | Human | NONE | 1,007,068 | 2,189 | 0.22 | 0.00 | 0 | 2 | ERR1081342,ERR1730377 |
| PDB14-201SH | South Sudan | Human | NUC | 11,201,696 | 9,920,172 | 88.56 | 12.05 | 10 | 2 | ERR1081344,ERR1243214 |
| Dmed10-14_S_H | South Sudan | Human | NUC | 138,443,686 | 24,876,967 | 17.97 | 23.95 | 19 | 4 | ERR273907,ERR273929,ERR563493,ERR563499 |
| 2014-1ChD | Chad | Dog | NUC | 25,387,772 | 15,168,391 | 59.75 | 18.49 | 14 | 2 | ERR1081328,ERR1243211 |
| 2014-2ChD | Chad | Dog | NUC | 118,848,030 | 59,278,247 | 49.88 | 71.60 | 61 | 2 | ERR1081357,ERR1243221 |
| 2014-3ChD | Chad | Dog | NUC | 20,821,588 | 13,348,157 | 64.11 | 16.12 | 14 | 2 | ERR1081358,ERR1243222 |
| 2014-4ChD | Chad | Dog | NUC | 34,795,436 | 17,975,620 | 51.66 | 21.76 | 19 | 2 | ERR1081359,ERR1243223 |
| 2014-5ChD | Chad | Dog | MIT | 429,444 | 55,993 | 13.04 | 0.08 | 0 | 1 | ERR1081346 |
| 2014-6ChD | Chad | Dog | MIT | 680,944 | 203,121 | 29.83 | 0.29 | 0 | 1 | ERR1081347 |
| 2014-7ChD | Chad | Dog | MIT | 665,416 | 216,817 | 32.58 | 0.31 | 0 | 1 | ERR1081348 |
| 2014-8ChD | Chad | Dog | NONE | 338,870 | 40,552 | 11.97 | 0.06 | 0 | 1 | ERR1081349 |
| 2015-1ChD | Chad | Dog | NUC | 24,776,440 | 14,464,333 | 58.38 | 17.47 | 13 | 2 | ERR1081350,ERR1243215 |
| 2015-2ChD | Chad | Dog | MIT | 8,900,594 | 7,848,792 | 88.18 | 9.51 | 8 | 2 | ERR1081351,ERR1243216 |
| 2015-3ChD | Chad | Dog | NUC | 11,763,796 | 10,321,366 | 87.74 | 12.54 | 11 | 2 | ERR1081352,ERR1243217 |
| 2015-4ChD | Chad | Dog | NUC | 33,738,410 | 10,758,127 | 31.89 | 13.03 | 11 | 2 | ERR1081326,ERR1243209 |
| 2015-5ChD | Chad | Dog | NUC | 11,309,548 | 9,933,132 | 87.83 | 12.06 | 10 | 2 | ERR1081327,ERR1243210 |
| 2015-6ChD | Chad | Dog | NUC | 17,397,832 | 11,787,265 | 67.75 | 14.24 | 13 | 2 | ERR1081353,ERR1243218 |
| 2015-7ChD | Chad | Dog | MIT | 324,328 | 198,454 | 61.19 | 0.29 | 0 | 1 | ERR1081354 |
| 2015-8ChD | Chad | Dog | MIT | 8,057,292 | 6,838,667 | 84.88 | 8.27 | 7 | 2 | ERR1081355,ERR1243219 |
| BDB06-5a | Cote d'Ivoire | Human | NUC | 89,024,662 | 67,376,292 | 75.68 | 65.70 | 55 | 5 | ERR460381,ERR563547,ERR563553,ERR563559,ERR563564 |
| BDB01-16a | Togo | Human | NONE | 1,800,622 | 1,522 | 0.08 | 0.00 | 0 | 1 | ERR460379 |
| BDB01-16b | Togo | Human | NONE | 1,798,440 | 1,682 | 0.09 | 0.00 | 0 | 1 | ERR460380 |
| Dmed06-7_N_H | Niger | Human | NONE | 9,731,926 | 25,777 | 0.26 | 0.02 | 0 | 2 | ERR273906,ERR273928 |
| Dmed11-1_Ch_H | Chad | Human | NUC | 60,468,586 | 50,606,117 | 83.69 | 48.74 | 39 | 6 | ERR273901,ERR273923,ERR563491,ERR563497,ERR563525,ERR563536 |
| Dmed112-40_Ch_D | Chad | Dog | MIT | 16,826,492 | 535,336 | 3.18 | 0.52 | 0 | 2 | ERR273914,ERR273936 |
| Dmed12-12_Ch_H | Chad | Human | NONE | 4,146,036 | 15,934 | 0.38 | 0.02 | 0 | 2 | ERR273902,ERR273924 |
| Dmed12-15_Ch_H | Chad | Human | NONE | 4,465,058 | 3,230 | 0.07 | 0.00 | 0 | 2 | ERR273903,ERR273925 |
| Dmed12-34_E_H | Ethiopia | Human | MIT | 12,117,156 | 1,727,610 | 14.26 | 1.66 | 1 | 2 | ERR273908,ERR273930 |
| Dmed12-35_Ch_H | Chad | Human | NONE | 11,603,600 | 313,203 | 2.7 | 0.30 | 0 | 2 | ERR273904,ERR273926 |
| Dmed12-37_Ch_D | Chad | Dog | MIT | 14,276,312 | 55,830 | 0.39 | 0.05 | 0 | 4 | ERR273912,ERR273934,ERR319496,ERR319497 |
| Dmed12-38_Ch_D | Chad | Dog | NUC | 69,686,582 | 61,063,480 | 87.63 | 58.83 | 52 | 6 | ERR273913,ERR273935,ERR563495,ERR563501,ERR563529,ERR563540 |
| Dmed12-42_Ch_D | Chad | Dog | MIT | 15,437,512 | 527,224 | 3.42 | 0.51 | 0 | 2 | ERR273915,ERR273937 |
| Dmed12-43_Ch_D | Chad | Dog | MIT | 15,124,806 | 1,363,831 | 9.02 | 1.31 | 0 | 2 | ERR273916,ERR273938 |
| Dmed12-49_M_H | Mali | Human | MIT | 11,720,346 | 21,964 | 0.19 | 0.02 | 0 | 2 | ERR273909,ERR273931 |
| Dmed12-58_Ch_H | Chad | Human | NUC | 255,743,466 | 216,521,130 | 84.66 | 208.59 | 180 | 8 | ERR273905,ERR273927,ERR563492,ERR563498,ERR563526,ERR563537,ERR563560,ERR563565 |
| Dmed12-60_M_H | Mali | Human | MIT | 1,886,708 | 1,437,002 | 76.16 | 1.38 | 1 | 2 | ERR273910,ERR273932 |
| Dmed13-8_Ch_D | Chad | Dog | MIT | 193,521,152 | 31,504,467 | 16.28 | 30.60 | 10 | 3 | ERR349721,ERR563503,ERR563514 |
| DmedGCW_G_H | Ghana | Human | NUC | 51,467,850 | 44,327,136 | 86.13 | 42.71 | 37 | 6 | ERR273911,ERR273933,ERR563494,ERR563500,ERR563528,ERR563539 |
| PDB09-5a | Niger | Human | MIT | 952,294 | 529,410 | 55.59 | 0.76 | 0 | 1 | ERR460384 |
| PDB09-5b | Niger | Human | NONE | 1,654,502 | 2,611 | 0.16 | 0.00 | 0 | 1 | ERR460385 |
| PDB12-58_adF_Ch_H | Chad | Human | MIT | 1,855,966 | 1,017,293 | 54.81 | 1.47 | 1 | 1 | ERR349722 |
| PDB12-58a_L1MF_Ch_H | Chad | Human | NUC | 78,014,152 | 66,426,989 | 85.15 | 64.98 | 58 | 5 | ERR349723,ERR563504,ERR563515,ERR563530,ERR563541 |
| PDB12-60_adF_Mali_H | Mali | Human | NUC | 43,539,932 | 28,816,385 | 66.18 | 27.92 | 23 | 5 | ERR349724,ERR563505,ERR563516,ERR563531,ERR563542 |
| PDB12-67_adF_Ch_D | Chad | Dog | NUC | 139,667,262 | 100,037,721 | 71.63 | 97.62 | 85 | 5 | ERR349729,ERR563508,ERR563519,ERR563532,ERR563543 |
| PDB12-67_L1MF_Ch_D | Chad | Dog | MIT | 819,306 | 634,946 | 77.5 | 0.92 | 1 | 1 | ERR349730 |
| PDB12-70a_L1MF_Ch_H | Chad | Human | NUC | 92,134,304 | 23,316,394 | 25.31 | 22.64 | 14 | 3 | ERR349726,ERR563506,ERR563517 |
| PDB12-70b_adF_Ch_H | Chad | Human | NONE | 402,610 | 187,091 | 46.47 | 0.27 | 0 | 1 | ERR349725 |
| PDB13-17_adF_Ch_H | Chad | Human | MIT | 1,510,120 | 432,055 | 28.61 | 0.62 | 0 | 1 | ERR349727 |
| PDB13-17_L1MF_Ch_H | Chad | Human | NUC | 23,046,238 | 15,617,912 | 67.77 | 15.47 | 13 | 3 | ERR349728,ERR563507,ERR563518 |
| PDB13-19_adF_Ch_D | Chad | Dog | NUC | 88,741,138 | 76,943,100 | 86.71 | 75.18 | 65 | 5 | ERR349731,ERR563509,ERR563520,ERR563533,ERR563544 |
| PDB13-27b1_adF_Ch_D | Chad | Dog | NUC | 139,764,890 | 95,458,521 | 68.3 | 92.70 | 74 | 5 | ERR349732,ERR563510,ERR563521,ERR563534,ERR563545 |
| PDB13-27c1_adF_Ch_D | Chad | Dog | MIT | 2,157,112 | 782,842 | 36.29 | 1.13 | 1 | 1 | ERR349733 |
| PDB13-27c1_L1MF_Ch_D | Chad | Dog | MIT | 15,484,644 | 10,348,781 | 66.83 | 10.26 | 8 | 3 | ERR349734,ERR563511,ERR563522 |
| PDB13-36_AdFem_Eth | Ethiopia | Human | NUC | 100,290,004 | 66,446,625 | 66.25 | 64.50 | 55 | 5 | ERR349735,ERR563512,ERR563523,ERR563535,ERR563546 |
| PDB13-36_L1mix_Eth | Ethiopia | Human | MIT | 1,023,952 | 637,308 | 62.24 | 0.92 | 1 | 1 | ERR349736 |
| PDB13-38_AdFem_Eth | Ethiopia | Human | MIT | 2,162,982 | 1,208,094 | 55.85 | 1.75 | 1 | 1 | ERR349737 |
| PDB13-38_L1mix_Eth | Ethiopia | Human | MIT | 10,231,108 | 5,883,801 | 57.51 | 5.87 | 5 | 3 | ERR349738,ERR563513,ERR563524 |
| PDB13-46 | Chad | Cat | NUC | 59,685,340 | 51,250,126 | 85.87 | 50.15 | 43 | 5 | ERR460386,ERR563548,ERR563554,ERR563561,ERR563566 |
| PDB13-78-Ad | Ethiopia | Baboon | NUC | 131,493,342 | 94,794,894 | 72.09 | 92.26 | 77 | 3 | ERR460387,ERR563549,ERR563555 |
| PDB13-78-L1 | Ethiopia | Baboon | NUC | 245,165,212 | 139,723,590 | 56.99 | 135.99 | 121 | 3 | ERR460389,ERR563550,ERR563556 |
| PDB13-92-Ad | Ethiopia | Dog | NUC | 59,912,414 | 52,943,803 | 88.37 | 51.67 | 43 | 5 | ERR460388,ERR563527,ERR563538,ERR563562,ERR563567 |
| PDB13-92-L1 | Ethiopia | Dog | NUC | 122,349,456 | 43,334,484 | 35.42 | 42.10 | 37 | 5 | ERR460390,ERR563551,ERR563557,ERR563563,ERR563568 |
| PDB14-100CH | Chad | Human | MIT | 8,469,766 | 6,871,657 | 81.13 | 8.32 | 7 | 2 | ERR1081332,ERR1243213 |
| PDB14-135CH | Chad | Human | NUC | 17,636,172 | 12,076,788 | 68.48 | 14.66 | 13 | 2 | ERR1081331,ERR1243212 |
| PDB14-207CH | Chad | Human | NONE | 219,034 | 1,666 | 0.76 | 0.00 | 0 | 2 | ERR1081362 |
| PDB14-209CH | Chad | Human | NONE | 465,120 | 15,711 | 3.38 | 0.02 | 0 | 1 | ERR1081322 |
| PDB14-210CH | Chad | Human | NONE | 822,112 | 1,090 | 0.13 | 0.00 | 0 | 3 | ERR1081361 |
| PDB14-22CH | Chad | Human | NONE | 377,076 | 1,623 | 0.43 | 0.00 | 0 | 1 | ERR1081336 |
| PDB14-223CH | Chad | Human | NUC | 12,766,930 | 11,176,393 | 87.54 | 13.55 | 11 | 2 | ERR1081325,ERR1243208 |
| PDB14-24CH | Chad | Human | NUC | 26,045,970 | 15,618,401 | 59.96 | 18.88 | 16 | 2 | ERR1081319,ERR1243206 |
| PDB14-240MH | Mali | Human | MIT | 374,388 | 261,532 | 69.86 | 0.38 | 0 | 1 | ERR1081341 |
| PDB14-253MH | Mali | Human | MIT | 608,986 | 296,201 | 48.64 | 0.43 | 0 | 1 | ERR1081340 |
| PDB14-269MH | Mali | Human | NONE | 599,264 | 376,797 | 62.88 | 0.54 | 0 | 1 | ERR1081338 |
| PDB14-278EH | Ethiopia | Human | MIT | 834,286 | 82,418 | 9.88 | 0.12 | 0 | 1 | ERR1081337 |
| PDB14-279CH | Chad | Human | NONE | 1,091,738 | 7,128 | 0.65 | 0.01 | 0 | 1 | ERR1081324 |
| PDB14-283CH | Chad | Human | MIT | 778,050 | 339,157 | 43.59 | 0.49 | 0 | 1 | ERR1081323 |
| PDB14-68CH | Chad | Human | MIT | 286,718 | 184,537 | 64.36 | 0.27 | 0 | 1 | ERR1081333 |
| PDB14-69CH | Chad | Human | NONE | 987,276 | 132,848 | 13.46 | 0.19 | 0 | 1 | ERR1081364 |
| PDB15-18CH | Chad | Human | MIT | 17,468,312 | 10,416,113 | 59.63 | 12.57 | 10 | 2 | ERR1081360,ERR1243224 |
| PDB15-24CH | Chad | Human | NONE | 306,614 | 97,299 | 31.73 | 0.14 | 0 | 1 | ERR1081330 |
| PDB15-46CH | Chad | Human | NONE | 151,762 | 2,543 | 1.68 | 0.00 | 0 | 1 | ERR1081329 |
| REF | Ghana | Human | NUC | 300,273,364 | 284,679,336 | 94.81 | 274.25 | 244 | 1 | ERR066175 |
| ferret A female | N/A* | N/A | N/A | 323,962,030 | 91,896,737 | 90.1 | 424.30 | 391 | 1 | ERR1945309 |
| ferret B male | N/A* | N/A | N/A | 316,685,164 | 274,866,059 | 86.79 | 399.50 | 365 | 1 | ERR1945310 |
| ferret B female | N/A* | N/A | N/A | 293,016,784 | 261,298,996 | 89.18 | 379.81 | 346 | 1 | ERR1945311 |

*parasites originated from an infected dog from Chad
