# Supplementary material for: Population genomic evidence that human and animal infections in Africa come from the same populations of Dracunculus medinensis
Source: PLoS Negl Trop Dis. 2020 Nov 30;14(11):e0008623. doi: 10.1371/journal.pntd.0008623 (PMC7728184; doi:10.1371/journal.pntd.0008623)
Supplement: S2 Table — Reads and mapping statistics are for the sum across all sequenced libraries and lanes. ENA = European Nucleotide Archive. (DOCX) [file pntd.0008623.s009.docx]

| sample name | country | host | species | total reads | reads mapping | percent mapping | mean coverage | median coverage | number of lanes | ENA accession numbers |
| --- | --- | --- | --- | --- | --- | --- | --- | --- | --- | --- |
| Din88-31 | USA |  | insignis | 251825454 | 56729856 | 22.5 | 33.37 | 12 | 4 | ERR273919, ERR273941, ERR563496, ERR563502 |
| Din88-31L1 | USA |  | insignis | 147069250 | 32767530 | 22.3 | 21.43 | 8 | 4 | ERR273922, ERR273944, ERR563552, ERR563558 |
| Dlut | Canada |  | lutrae | 87800942 | 2292804 | 2.6 | 2.14 | 1 | 2 | ERR1081356, ERR1243220 |
