# Supplementary material for: Population genomic evidence that human and animal infections in Africa come from the same populations of Dracunculus medinensis
Source: PLoS Negl Trop Dis. 2020 Nov 30;14(11):e0008623. doi: 10.1371/journal.pntd.0008623 (PMC7728184; doi:10.1371/journal.pntd.0008623)
Supplement: S3 Table — Values are means of the posterior distribution and 95% highest posterior density confidence intervals. Θ values are effective population size estimates and τ are divergence time estimates. East+Chad and Africa representing the two ancestral populations, as shown on Fig 4. (DOCX) [file pntd.0008623.s010.docx]

| prior | Θ_W. Africa_ | Θ_Chad_ | Θ_E. Africa_ | Θ_East+Chad_ | Θ_Africa_ | 𝜏_East+Chad_ | 𝜏_Africa_ |
| --- | --- | --- | --- | --- | --- | --- | --- |
| 𝛼=10^-8^ | 21,538  (18,888-24,184) | 31,087  (20,496-40,550) | 7,849  (5024-10502) | 336,776  (251697-426576) | 112,057  (106969-117231) | 4,106  (2572-5542) | 20,643  (18326-22889) |
| 𝛼=10^-7^ | 21,625  (19160-24188) | 28,361  (18744-36941) | 7,151  (4756-9323) | 349,031  (252008-463045) | 112,494  (107749-117749) | 3,729  (2347-4809) | 20,760  (18660-22996) |
| 𝛼=10^-6^ | 21,147  (18633-23594) | 28,192  (18103-36990) | 7,370  (4603-9464) | 394,842  (273224-504162) | 112,903  (108006-118058) | 3,841  (2358-5080) | 20,398  (18047-22869) |
| 𝛼=10^-5^ | 21,563  (18831-24496) | 29,894  (20177-39670) | 7,617  (4998-10102) | 350,866  (253046-471265) | 112,844  (107483-118213) | 4,014  (2572-5421) | 20,612  (18150-23222) |
| 𝛼=10^-4^ | 21,233  (18420-23886) | 32,283  (22994-41750) | 8,153  (5596-10490) | 340,002  (244403-437895) | 112,930  (107743-118174) | 4,305  (2895-5559) | 20,319  (17666-22705) |
